# Sound Transmission Loss of a Sandwich Plate with Adjustable Core Layer Thickness

**DOI:** 10.3390/ma13184160

**Published:** 2020-09-18

**Authors:** Tom Ehrig, Martin Dannemann, Ron Luft, Christian Adams, Niels Modler, Pawel Kostka

**Affiliations:** 1 Institute of Lightweight Engineering and Polymer Technology, Technische Universität Dresden, 01069 Dresden, Germany; martin.dannemann@tu-dresden.de (M.D.); ron.luft@tu-dresden.de (R.L.); niels.modler@tu-dresden.de (N.M.); pawel.kostka@tu-dresden.de (P.K.); 2 Department of Mechanical Engineering, System Reliability, Adaptive Structures, and Machine Acoustics SAM, Technical University of Darmstadt, 64289 Darmstadt, Germany; christian.adams@sam.tu-darmstadt.de

**Keywords:** sound transmission loss, semi-active damping, sandwich panel, morphing structure, compressible constrained layer damping, composite materials

## Abstract

Compressible Constrained Layer Damping (CCLD) is a novel, semi-active, lightweight-compatible solution for vibration mitigation based on the well-known constrained layer damping principle. The sandwich-like CCLD set-up consists of a base structure, a constraining plate, and a compressible open-cell foam core in between, enabling the adjustment of the structure’s vibration behaviour by changing the core compression using different actuation pressures. The aim of the contribution is to show to what degree, and in which frequency range the acoustic behaviour can be tuned using CCLD. Therefore, the sound transmission loss (TL), as an important vibro-acoustic index, is determined in an acoustic window test stand at different actuation pressures covering a frequency range from 0.5 to 5 kHz. The different actuation pressures applied cause a variation of the core layer thickness (from 0.9 *d*_0_ to 0.3 *d*_0_), but the resulting changes of the stiffness and damping of the overall structure have no significant influence on the TL up to approximately 1 kHz for the analysed CCLD design. Between 1 kHz and 5 kHz, however, the TL can be influenced considerably well by the actuation pressure applied, due to a damping-dominated behaviour around the critical frequency.

## 1. Introduction

Fibre-reinforced plastics (FRP) are increasingly used in everyday products. In particular, the use of FRP with glass and carbon fibres as reinforcing materials has spread from the aerospace sector, via automotive engineering and medical technology, to general mechanical engineering due to their excellent mechanical properties at low weight [1]. Considering that mass, stiffness and damping are coupled design variables of FRP, the lightweight oriented design often causes a problematic vibration susceptibility of the developed components. The consequences of component vibrations range from the subjective feeling of a low-quality component, to actual noise exposure to the failure of the component under high dynamic loads. In order to avoid potential environmental and health issues as well as damage-relevant or function-impairing amplitudes caused by vibrations, systems for health monitoring [2,3] and solutions for vibration mitigation were developed. The latter can be passive, semi-active or active damping treatments [4,5,6,7,8,9,10,11,12,13]. Most of these damping concepts achieve very good results. However, the overall implementation costs of semi-active, and especially active, damping systems are still high. Moreover, the additional mass of structure-integrated and peripheral hardware is a major drawback for many applications [14]. As part of their current research, the authors are pursuing a novel approach to combine the two requirements—lightweight design and efficient adjustable damping—for a new generation of lightweight structures.

### 1.1. Operating Principle of Compressible Constrained Layer Damping

**Figure 1:**
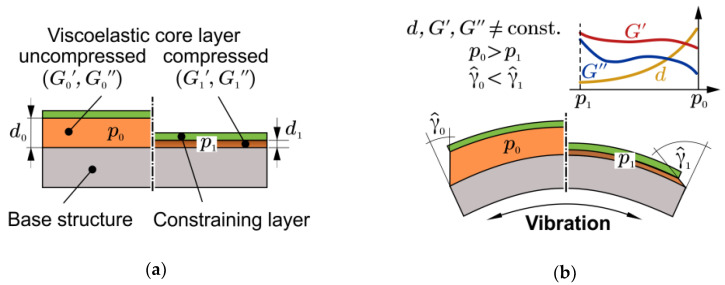
Illustration of the Compressible Constrained Layer Damping (CCLD) principle: (**a**) set-up without bending; (**b**) under vibration.

For the adaptation of the dynamic behaviour, the authors have already proposed an original concept of Compressible Constrained Layer Damping (CCLD) in previous publications [15,16,17,18]. CCLD is an extension of the well-known Constrained Layer Damping (CLD) design, where the usually incompressible viscoelastic damping layer, is replaced by a compressible one, which is able to undergo compression and expansion in thickness direction. The adaptation principle of CCLD is therefore based on the pressure-controlled thickness variation of the viscoelastic core layer (Figure 1). While the storage and loss shear modulus of the viscoelastic layer G′ and G′′, which affect the damping performance significantly, as well as the thickness of the viscoelastic layer are constant in CLD, they depend on the actuation pressure p in the case of CCLD.

The adjustable viscoelastic properties of the core layer, as well as the altering of the shear deformation amplitude, $\hat{\gamma }$, by variation of the core layer thickness, *d*, finally lead to a simple yet efficient adaptation of the dynamic behaviour of the overall system. The actual damping mechanism is based, as in CLD, on the shear deformation induced within the constrained damping layer. The actuation pressure could be provided, e.g., by hydraulic fluid, compressed air, or vacuum. However, in the present paper, only vacuum actuation will be considered. 

In a previous publication [15], the authors have analysed the mobility (ratio of vibration velocity to excitation force) of a composite plate with CCLD in a lower acoustical frequency range. In this case of force excitation, which is relevant, e.g., in mechanical engineering, a complex, frequency-dependent control of the CCLD can significantly reduce the surface velocities, and thus the radiated sound power. In the present contribution, the sound absorbing ability of a CCLD-equipped plate is investigated.

### 1.2. Sound Transmission Loss of Sandwich Structures

The sound transmission loss (TL) is an important vibro-acoustic index to determine how well structures insulate sound. The TL is defined as the ratio of incident to transmitted sound power. There are a number of methods and publications for calculating the TL of FRP and sandwich structures, which are comprehensively reviewed by D’Alessandro et al. [19] and by Isaac et al. [20]. The studies of Dym and Lang [21] are of particular interest, as they describe the effects of skin thickness variation (and their ratio) and core stiffness variation in great detail. All of these models and experimental data show that the influence of the core layer properties (material, thickness, loss factor) on the TL is strongly dependent on the frequency range, especially if it is below or above the critical frequency. The influence of the foam core’s shear stiffness on the critical frequency is modelled numerically in [22]. According to these calculations, a decrease of the critical frequency is expected with an increasing shear stiffness of the foam core. From the authors’ own investigations [23], it is known that the correlation between the shear property changes of the foam core material and increasing compression is strongly non-linear. Furthermore, there is no linear correlation between skin-to-core thickness ratio and TL [24]. According to the work of Liu et al. [25,26], it can be expected that the TL improves with decreasing internal pressure. However, the publication of Du et al. [27] indicates that the variation of thickness and shear modulus of the viscoelastic core layer has no significant influence on the TL. All these results lead to the conclusion that it is hard to estimate the TL and the critical frequency as functions of actuation pressure in advance, although it is to be expected that the damping effect of the CCLD will be mainly present in the stiffness-dominated region, as well as in the damping-dominated region around and above the critical frequency.

The aim of the contribution is the experimental investigation of the acoustic effects of the CCLD concept. In particular, the change of TL and the shift of the critical frequency through varying the actuation pressure will be considered. The unique feature of the CCLD treatment in the acoustical context is that the geometric dimensions of the test structure remain constant (except for the core layer thickness *d*), as does the total mass of the structure. Simultaneously, the shear deformation kinematics on the structural level are changed by adjusting the actuation pressure, as well as damping and stiffness, of the viscoelastic core material, resulting in altered acoustic properties.

## 2. Materials and Test Set-up Configuration

### 2.1. Materials

The sandwich-like multilayer CCLD set-up consists of three layers: the base structure; the constraining layer; and the constrained viscoelastic core layer. It is an asymmetric sandwich structure, as the top layer is only one tenth of the thickness of the base structure, and, in addition, has high shear and tensile but low flexural stiffness.

A carbon fibre-reinforced plastics (CFRP) plate with a length of $a_{b}=$1100 mm, a width of $b_{b}=$ 700 mm, and a thickness of $d_{b}=$ 2 mm was used as base structure. The plate was made of seven prepreg layers (Co. Carboplast, Werne, Germany, HTA fibres and epoxy resin, hot-pressing process) with a [0/90,0_2_,90,0_2_,0/90] lay-up resulting in low intrinsic structural damping. The viscoelastic core layer consisted of the open-cell polyurethane foam Confor CF-47M (Co. Aearo Technologies LLC, Indianapolis, United States of America). The size of the viscoelastic core layer was set to be 880 mm × 580 mm to ensure complete CCLD coverage of the sound-exposed area by overlapping the dimensions of the acoustic window (860 mm × 560 mm). The thickness *d*_0_ in the uncompressed state was equal to 10 mm. A CFRP sheet (Co. R&G, Waldenbuch, Germany) with the dimensions of 880 mm × 580 mm and a thickness of 0.2 mm was used as constraining layer. The CFRP sheet, which is produced by the technique of hot-pressing, consists of just one layer of HT carbon fibre prepreg (grammage 200 g/m^2^, twill 2/2 weave) and an epoxy matrix. Table 1 contains the materials used and their main properties.

### 2.2. CCLD Test Set-up Configuration

**Figure 2:**
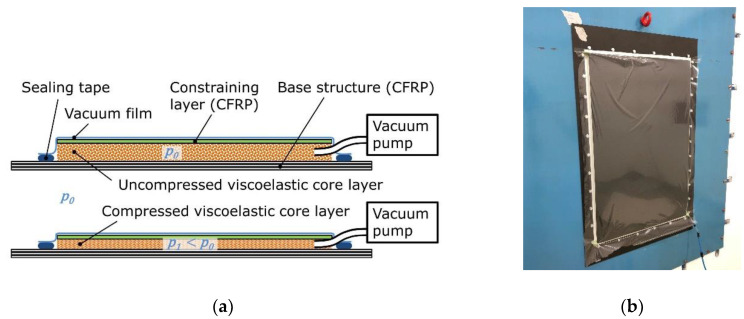
Lay-up of the base structure with CCLD treatment: (**a**) without actuation pressure (**top**) and with applied actuation pressure (**bottom**); (**b**) Test structure in the window test stand, receiver room.

For the acoustic measurements, the CCLD treatment was set up as shown in Figure 2. The base structure, the damping layer, and the constraining layer were stacked on top of each other, while vacuum-sealing was assured by using a vacuum film and vacuum sealing tape. The individual layers were not glued together to avoid introducing another viscoelastic layer and to eliminate further phenomena, such as curing of the adhesive in the open-cell pores. The set-up was kept in position by the tightly applied vacuum film, and later fixed by the vacuum pressure enabling coupling of the layers through friction forces. The applied actuation pressure was generated by a vacuum pump and adjusted with a valve to achieve the predefined core layer thicknesses (Table 2). After setting the desired actuation pressure and a waiting time of >1 min an evenly distributed foam compression was achieved over the entire surface of the plate. Only at $p_{1}=$ −16 kPa (corresponding to $0.3\;d_{0}$) a slightly smaller compression of the foam was observed in the edge area up to max. 20 mm from the edges. The base structure with the applied CCLD treatment (hereinafter referred to as ’test structure’ for the sake of simplicity) was then rigidly clamped in the acoustic window test stand using screws on all of its edges with the CCLD treatment facing the receiver room. The size of the base structure and the dimensions of the CCLD treatment were carefully chosen so that there were no air gaps or additional acousto-mechanical bridges in the test structure.

### 2.3. Sound Transmission Loss Measurements

**Figure 3:**
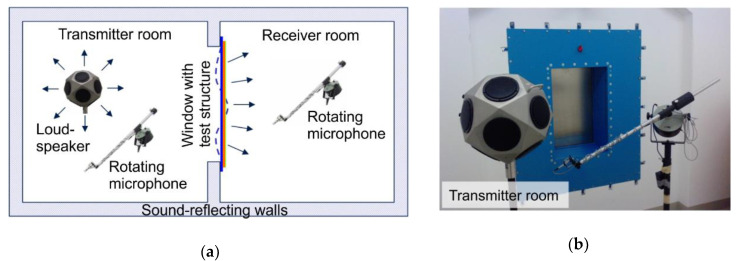
(**a**) Set-up for the sound transmission loss measurement; (**b**) transmitter room of the acoustic test stand.

The TL was determined in an acoustic window test stand consisting of a transmitter room and a receiver room. Both rooms were connected by a window with the dimensions of 860 mm × 560 mm, which contained the test structure (Figure 3).

A diffuse sound field in the transmitter room was used to acoustically excite the test structure, which radiated sound into the receiver room. During excitation, the spatially and temporally averaged sound pressure was measured in the transmitter as well as in the receiver room using rotating microphones. The same set of microphone measuring positions was used for each applied actuation pressure. Based on those sound pressure measurements, the TL was calculated using the standardized procedure [28]. In accordance with the standards [28,29] and in order to ensure that a diffuse sound field was established within the given room dimensions, the lower limit of the measuring range was set at 0.5 kHz. According to standard [29], the upper limit of the measuring range is 3.15 kHz. Since our analytical calculation (see Section 4.1.) predicted an $f_{c}$ at about 4.8 kHz, we chose the extended measuring range defined in [29], which specifies 5 kHz as the upper limit of the measuring range.

## 3. Sound Transmission Loss Calculation of the Base Structure

The TL of an acoustic wave that incidents an infinite plate under the angle ϕ with respect to the normal direction of a plate’s surface can be written as [30]

(1) $$\mathrm{TL}=10\log_{10}\left(\frac{{\left(\frac{2\rho_{L}c_{L}}{\cos\left(\phi \right)}+\frac{B_{b}}{2\pi f}\eta_{b}{\left(\frac{2\pi f}{c_{L}}\sin\left(\phi \right)\right)}^{4}\right)}^{2}+{\left(2\pi f\rho_{b}d_{b}-\frac{B_{b}}{2\pi f}{\left(\frac{2\pi f}{c_{L}}\sin\left(\phi \right)\right)}^{4}\right)}^{2}}{{\left(\frac{2\rho_{L}c_{L}}{\cos\left(\phi \right)}\right)}^{2}}\right)\mathrm{dB},$$

where $\rho_{L}$ is the air density, $c_{L}$ is the speed of sound, $\eta_{b}$ is the base structure’s material loss factor, $\rho_{b}$ is the mass density of the base structure, and f is the frequency. According to [31], the equivalent flexural stiffness of an orthotropic plate yields

(2) $$B_{b}={\left(\frac{\sqrt{B_{b,0{}^{\circ}}}}{2}+\frac{\sqrt{B_{b,90{}^{\circ}}}}{2}\right)}^{2},$$

With

(3) $$B_{b,0{}^{\circ}}=\frac{E_{b,0{}^{\circ}}d_{b}^{\;3}}{12\left(1-\mu_{b}^{\;2}\right)}\;\;\;\;\;\;\;\mathrm{and}\;\;\;\;\;\;\;B_{b,90{}^{\circ}}=\frac{E_{b,90{}^{\circ}}d_{b}^{\;3}}{12\left(1-\mu_{b}^{\;2}\right)}.$$

$E_{b,0{}^{\circ}}$ and $E_{b,90{}^{\circ}}$ denote the Young’s moduli in the plate’s 0° direction and 90° direction, respectively, and $\mu_{b}$ denotes Poisson’s ratio. Although Equation (1) assumes that the plate is infinite, its results are compared to the TL measurements of the base structure, which has finite dimensions. It is a first starting point to check and better understand the measurement results. Equation (1) is easy to implement, which is an advantage over more complex models such as finite element models. An improved model for TL calculations should take into account the boundary conditions of the base structure and the acoustic properties of the transmitter room and the receiver room. These aspects are neglected in Equation (1) and can be considered in numerical models only, which are beyond the scope of this paper. It is well known from the literature [14,19,21] that the TL can be divided into three characteristic frequency ranges. At frequencies up to $2f_{r}$, the TL is stiffness-dominated, where $f_{r}$ denotes the fundamental frequency. From $2f_{r}$ through $f_{c}/2$ the TL is mass-dominated, where $f_{c}$ denotes the critical frequency. Above the critical frequency the TL is damping-dominated. The following equations are used in this paper to estimate these frequency ranges for the base structure. As the base structure was clamped on all edges, its fundamental frequency could be estimated by [32]

(4) $$f_{r}\approx \frac{6}{\pi }\sqrt{\frac{7}{2}\left(\frac{1}{a_{b}^{\;4}}+\frac{4}{7}\frac{1}{a_{b}^{\;2}b_{b}^{\;2}}+\frac{1}{b_{b}^{\;4}}\right)}\sqrt{\frac{B_{b}}{\rho_{b}d_{b}}}\;.$$

The critical frequency $f_{c}$ of the base structure reads [32]

(5) $$f_{c}=\frac{c_{L}^{\;2}}{2\pi }\sqrt{\frac{\rho d_{b}}{B_{b}}}\;.$$

## 4. Results and Discussion

This section first characterizes the base structure and compares the measured results to those from TL calculations of an infinite plate. Secondly, the results of the test structure are discussed.

### 4.1. Base Structure

**Figure 4:**
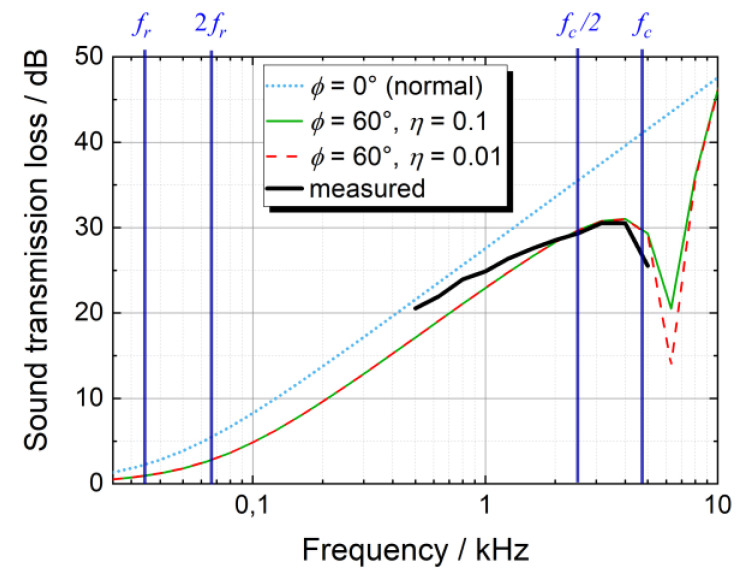
Measured and calculated sound transmission loss of the base structure versus frequency; vertical lines indicate the labelled frequencies.

**Figure 5:**
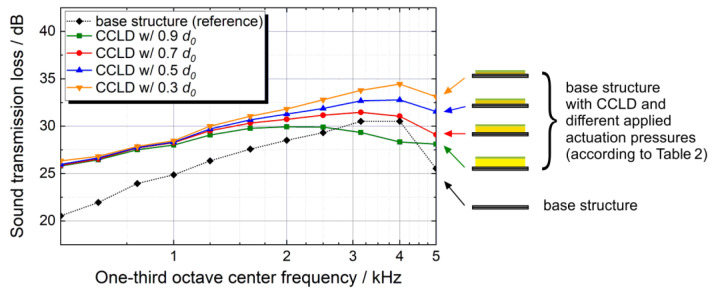
Sound transmission loss of the investigated test structure (base structure with CCLD treatment) at various core layer thicknesses (as result of different applied actuation pressures) compared to the transmission loss (TL) of the bare base structure. The repeat accuracy is <0.35 dB over the entire frequency range, therefore no error bars are displayed.

With the speed of sound $c_{L}=$ 343 m/s, the air’s density $\rho_{L}=$ 1.2 kg/m³, and the base structure’s parameters ($a_{b}=$1100 mm, $b_{b}=$700 mm, $d_{b}=$2 mm, $\rho_{b}$ = 1550 kg/m³, $\mu_{b}$ = 0.35, $E_{b,0{}^{\circ}}$ = 62 GPa, and $E_{b,90{}^{\circ}}$ = 60 GPa [33]) the critical frequency yields 4.85 kHz according to Equation (5), and the fundamental frequency yields 33.3 Hz according to Equation (4). The mass-dominated region ranges from $2f_{r}=\;66.6\;$ Hz through $f_{c}/2=$ 2.42 kHz. Figure 4 shows the measured TL of the base structure (for more detail see also Figure 5) as well as the calculation results for three different incidence angles using Equation (1). Between 0.5 kHz and 0.8 kHz, the measured TL agrees well with the TL of an infinite plate if the sound waves incident in normal direction (ϕ= 0°). Between 1 kHz and 2 kHz, the measured TL increases by approximately 3 dB, which is half of the increase that would have been expected by the mass law. Between $f_{c}/2=$ 2.42 kHz and $f_{c}=$ 4.85 kHz, the measured TL differs from the calculated one (ϕ= 0°) to a higher extent. In order to check whether the results in this frequency range can also be described by Equation (1), the incidence angle was iteratively increased until the calculated TL matched the measured one. At an angle of ϕ=60° the TL measurement and the TL calculation agree sufficiently well, see Figure 4. Thus, between $f_{c}/2$ and $f_{c}$ the measured TL equates more to that of an infinite plate that is excited by an acoustic wave under an angle of ϕ=60° than the TL of an infinite plate, where the acoustic waves incident in normal direction. In other words, the (finite) base structure behaves similarly to an infinite plate, where the acoustic wave incidents with an angle of ϕ=60°. These results suggest that all the aspects neglected in Equation (1), such as the base structure’s boundary conditions or the acoustic properties of transmitter room and receiver room, can be condensed into the incident angle ϕ. As such, the incident angle ϕ in Equation (1) could rather be seen as a hypothetical incident angle for an infinite plate than as the actual incident angle during the measurements. The loss factor of the base structure $\eta_{b}$ also affects the TL. As can be seen from Figure 4, this only affects the dip of the TL above the critical frequency. Since this dip is out of the measured frequency range, the loss factor of the base structure does not significantly affect the TL measurements performed in this paper. Consequently, the simple model of the TL shown in Equation (1) can qualitatively explain the measured TL if the incident angle ϕ is considered a parameter that covers all simplifications of the model with respect to the real measurement setup. Thus, the measured TL of the base structure is considered validated according to literature [14,19,21].

### 4.2. Base Structure with CCLD Treatment

Figure 5 shows the TL of the base structure as well as the TL of the test structure with various thicknesses. The frequency range from 0.5 kHz up to 1 kHz lies within the mass-controlled region, which is why there are no significant TL differences between the various compression levels (since the mass is always constant). With increasing frequency, the damping-dominated region becomes apparent and starting from $f_{c}/2\approx 2.4$ kHz, clear TL differences between the various compression levels are evident. It can be observed that the strongest compression (and thus the lowest core layer thickness) result in the highest TL.

The generally higher TL of the test structure at 0.3 *d*_0_ compared to the bare CFRP base structure over the whole frequency range (an increase of 6 dB at 0.5 kHz to 4 dB at 4 kHz) is a result of the approximately 30% mass increase due to the CCLD treatment and the higher damping of the more complex sandwich-like structure. At 4 kHz ($f_{c}/2<4\;\mathrm{kHz}<f_{c}$) the TL tends to decrease, which indicates that the TL becomes dominated by the damping.

A further benchmark for comparing the measurement results of the different core layer thicknesses is the weighted apparent sound reduction index *R***_w_**′. With this index, the measured frequency-dependent TL can be expressed by a single number. Using the procedure defined in ISO 717-1 [29], the measured TL spectra are shifted and the values of the reference curve at 0.5 kHz are determined. The results for the weighted apparent sound reduction index according to [29] are shown in Table 3.

While the differences between the various core layer thicknesses are rather obvious in the TL spectra above 1 kHz (Figure 5), the weighted apparent sound reduction index (Table 3) is hardly affected by the core layer thickness. As can be seen from Figure 5, at 0.5 kHz, i.e., the frequency at which the weighted apparent sound reduction index is obtained from the reference curve, the TL spectra are hardly affected by the core layer thickness. Consequently, the practical effect on the weighted apparent sound reduction index, which is commonly used in building acoustics, is small. Nevertheless, by a pressure-induced thickness reduction of the core layer, a significant increase of the TL can be achieved above 1 kHz. It should also be noted that the above-mentioned standard is a simplified procedure to determine a single value at 0.5 kHz, but the stiffness, mass and damping dominated regions are strongly dependent on the materials used and the dimensions of the specific CCLD set-up. Thus, with other configurations where the resonance frequency and critical frequency are lower, a stronger improvement of the weighted apparent sound reduction index may well be achieved.

## 5. Conclusions

The obtained results reveal that the acoustic properties of a structure could be altered using a CCLD treatment. In this publication, the focus was set on the TL, which can be increased using CCLD compared to the CFRP base structure without damping measures. The thickness variation of the core layer (from 0.9 *d*_0_ to 0.3 *d*_0_) set by different actuation pressures and the resulting stiffness and damping changes of the overall structure have no influence on the TL up to approximately 1 kHz for the analysed CCLD design. Between 1 kHz and 5 kHz (upper limit of the measured frequency range), however, it can be observed that the TL can be influenced considerably well by the actuation pressure applied, due to the fact that the TL is damping-dominated around the critical frequency and above. To further investigate the above-mentioned assumptions, measurements in a higher frequency range will be performed. This will also include measurements of the structural damping. In addition, a numerical model should also be built to further validate the measurements and to study the effect of other parameters of the CCLD design, such as thickness ratios or different viscoelastic materials. Finally, it should be noted that the differences between the TL of the various core layer thicknesses are rather obvious in the TL spectra. When assessed according to building acoustics standards using the weighted apparent sound reduction index, no practically relevant effects on the acoustic properties are observed with the analysed CCLD design, since the weighted apparent sound reduction index is obtained at 0.5 kHz, where the effect of the core layer thickness on the TL is small.

## Figures and Tables

**Table 1:** Compressible Constrained Layer Damping (CCLD) materials and their main properties.

Layer	Material	Manufacturer	Density *ρ*	Length *a*	Width *b*	Thickness *d*	Mass *m*	Mass per Unit Area
			kg/m³	mm	mm	mm	kg	kg/m²
Base structure	CFRP (HTA-CF, EP)	Carboplast	1550	1100	700	2	2.41	3.13
Visco-elastic layer	Viscoelastic foam Confor CF-47M	Aearo Technologies LLC	96	880	580	10	0.56	1.10
Constrain-ing layer	CFRP (HT-CF, EP)	R&G	1550	880	580	0.2	0.18	0.35

**Table 2:** Applied actuation pressure and corresponding core layer thickness (*d*_0_ = 10 mm).

Applied Actuation Pressure *p*_1_	−3.9 kPa	−5.9 kPa	−7.6 kPa	−16.0 kPa
Core layer thickness *d*_1_	0.9 *d*_0_	0.7 *d*_0_	0.5 *d*_0_	0.3 *d*_0_

**Table 3:** Weighted apparent sound reduction index (*R*_w_′) of the test structure at various core layer thicknesses compared to the bare CFRF base structure. The values for *R*_w_′ are determined with the stepwise adjustment (0.1 dB steps) specified in [29].

0.9 *d*_0_	0.7 *d*_0_	0.5 *d*_0_	0.3 *d*_0_	Base Structure
28.9 dB	29.4 dB	29.8 dB	30.1 dB	26.1 dB
